# 2,2’4,4’-Tetrabromodiphenyl Ether (PBDE-47) Modulates the Intracellular miRNA Profile, sEV Biogenesis and Their miRNA Cargo Exacerbating the LPS-Induced Pro-Inflammatory Response in THP-1 Macrophages

**DOI:** 10.3389/fimmu.2021.664534

**Published:** 2021-05-07

**Authors:** Valeria Longo, Alessandra Longo, Giorgia Adamo, Antonino Fiannaca, Sabrina Picciotto, Laura La Paglia, Daniele Romancino, Massimo La Rosa, Alfonso Urso, Fabio Cibella, Antonella Bongiovanni, Paolo Colombo

**Affiliations:** ^1^ Institute for Biomedical Research and Innovation, National Research Council of Italy (IRIB-CNR), Palermo, Italy; ^2^ High Performance Computing and Networking Institute, National Research Council of Italy (ICAR-CNR), Palermo, Italy

**Keywords:** flame retardant, THP-1 macrophage, small extracellular vesicle, microRNA, LPS, cytokines

## Abstract

The 2,2’4,4’-tetrabromodiphenyl ether (PBDE-47) is one of the most prominent PBDE congeners detected in the environment and in animal and human tissues. Animal model experiments suggested the occurrence of PBDE-induced immunotoxicity leading to different outcomes and recently we demonstrated that this substance can impair macrophage and basophil activities. In this manuscript, we decided to further examine the effects induced by PBDE-47 treatment on innate immune response by looking at the intracellular expression profile of miRNAs as well as the biogenesis, cargo content and activity of human M(LPS) macrophage cell-derived small extracellular vesicles (sEVs). Microarray and in silico analysis demonstrated that PBDE-47 can induce some epigenetic effects in M(LPS) THP-1 cells modulating the expression of a set of intracellular miRNAs involved in biological pathways regulating the expression of estrogen-mediated signaling and immune responses with particular reference to M1/M2 differentiation. In addition to the cell-intrinsic modulation of intracellular miRNAs, we demonstrated that PBDE-47 could also interfere with the biogenesis of sEVs increasing their number and selecting a *de novo* population of sEVs. Moreover, PBDE-47 induced the overload of specific immune related miRNAs in PBDE-47 derived sEVs. Finally, culture experiments with naïve M(LPS) macrophages demonstrated that purified PBDE-47 derived sEVs can modulate macrophage immune response exacerbating the LPS-induced pro-inflammatory response inducing the overexpression of the IL-6 and the MMP9 genes. Data from this study demonstrated that PBDE-47 can perturb the innate immune response at different levels modulating the intracellular expression of miRNAs but also interfering with the biogenesis, cargo content and functional activity of M(LPS) macrophage cell-derived sEVs.

## Introduction

Polybrominated diphenyl ethers (PBDEs) are a class of halogenated compounds that have emerged as a major environmental pollutant in recent years (1). This group of chemicals is used as flame-retardants (FRs) and are found in consumer goods such as electrical equipment, construction materials, coatings, textiles and polyurethane foam (furniture padding). Commercial production of PBDEs began in 1976 under various trade names, but they mainly consist of three commercial formulations known as penta- (DE-60F, DE-61, DE-62, and DE-71), octa- (DE-79), and deca-PBDE (DE-83R and Saytex 102E) (2). Similar in structure to several other environmental pollutants, PBDEs resist degradation in the environment and, for this reason, represent a threat to global health. Furthermore, unlike other reactive FRs which are mixed into plastic forming covalent bonds with the polymer matrix, PBDEs are additive flame retardants that are mixed with the polymers, creating greater potential to enter the environment from volatilization, leaching, or degradation (3). Due to their environmental persistence and bioaccumulation capability, the production of penta-BDE and octa-BDE were regulated in the USA and Europe, and in 2009 both classes of substances were included in the Stockholm Convention to promote their phase-out. In 2013, the USA introduced a restriction on deca-BDE production, and this class of substances was included in the list of persistent organic pollutants (POPs) at the Stockholm Convention in 2017. However, despite production restrictions, they still present a major global environmental risk in indoor contexts as well as outdoors in e-waste disposal and recycling activities (4) (United Nations Environment Program [UNEP], 2017), (European Parliament and of the Council, 2003), (U.S. Environmental Protection Agency [US EPA], 2010) (5).

To date, PBDEs have been detected in abiotic environments such as soil and water, as well as in several ecosystems (6). Furthermore, it has been demonstrated that PBDEs can enter the human body *via* inhalation, dermal contact, and ingestion, the latter being relevant in toddlers and children as they may spend much of their time on carpets, making them highly vulnerable to these contaminants (4). In addition, studies have demonstrated that PBDEs can bio-accumulate in human blood, breast milk, placenta, and fat tissues, as well as in animal tissues (7–10) entering the food chain (EFSA Panel, 2011).

PBDEs have been investigated for their toxicity in both *in vitro* and *in vivo* experimental set-ups. A large set of studies has reported that PBDEs might have multi system/organ toxicities in reproductive (11), endocrinological (12), immunological (13), pulmonary (14) and hepatic systems (15). In addition, as reviewed by Cai and coworkers (4), epidemiological studies have suggested an association between human exposure to PBDEs and changes in thyroid hormone regulation and neuropsychological functioning (16). Furthermore, the modulation of immune response has also been reported *in vivo* in aquatic species (17).

Recently, our research group has already addressed the question related to the ability of PBDE-47 to perturb the immune response, showing that this tetra-PBDE may impair the activities of cells involved in innate immunity, dampening the expression of macrophage pro-inflammatory cytokines (IL-1β, IL-6, and TNF-α) and genes involved in cell motility (MMP-12 and E-cadherin), and interfering with basophil activation (13).

The immune system is a very complex organization composed of different cell types spread all over the body and interacting with multiple organs through several communication systems. Immunological response is dependent on several mechanisms including cell-to-cell contacts, the release of a diverse array of soluble factors (i.e. cytokines and chemokines) and of extracellular vesicles (EVs) for communication with distant cells and organs. EVs are heterogeneous group of membranous nano-sized vesicles, originating from all cell types, having a long circulating half-life, and high specificity for target cells (18). In particular, small extracellular vesicles (sEVs, 30 to 150 nm diameter), also known as exosomes, mediate cell-to-cell communication by delivering their cargo content, such as DNA, mRNA, miRNA, lipids, and proteins into target or recipient cells (19). More recently, there has been increasing interest in the mechanism by which cells may exert their modulatory effects through the release of EVs (20, 21).

Macrophages are a key constituent of the innate immune response, involved in the first response to infection through their recognition of pathogen-associated molecular patterns (PAMPs) by pattern recognition receptors (PRRs), killing invading pathogens, phagocytizing dead and dying cells, responding to the pathogen invasion through cytokine release, and priming the adaptive immune response to generate antigen-specific response against infectious agents. Furthermore, macrophages are important for normal homeostasis, and prolonged activation signals leading to dysregulated macrophage activity can lead to pathological consequences (22).

In this manuscript, we analyzed the effect of PBDE-47 treatment on the intracellular profile of miRNAs expressed in the LPS-stimulated PMA differentiated THP-1 macrophage cell line. Furthermore, sEV features and their microRNAs cargo were analyzed in the same experimental set-up. Finally, purified sEVs from PBDE-treated THP-1 macrophages were tested in a culture assay using naïve M(0) macrophages, looking at the effect of those sEVs on the LPS inflammatory response induced in the PMA-differentiated THP-1 macrophage cell line.

## Materials and Methods

### Reagents

PBDE-47 (2,4,2’,4’-tetrabromodiphenyl ether) was purchased from Toronto Research Chemicals (ON, Canada) and dissolved at 25 mM in dimethyl sulfoxide (DMSO) (cat. n. D2650, Sigma-Aldrich, Milan, Italy) as a stock solution. The human monocytic leukemia THP-1 cell line (ECACC 88081201) was maintained in culture with RPMI 1640 medium (Gibco Life Technologies, Monza, Italy) supplemented with heat inactivated 10% Fetal Bovine Serum (FBS, Gibco Life Technologies, Monza, Italy) and 1% antibiotic (penicillin 5,000 U/mL, Streptomycin sulfate 5,000 µg/mL, Gibco Life Technologies, Monza, Italy).

### MiRNA Profiling in PBDE-47 Treated M(LPS) THP-1 Macrophages

To differentiate THP-1 monocytes into a M(0) macrophage phenotype, cells were treated with 200 nM phorbol 12-myristate-13-acetate (PMA, Sigma-Aldrich, Milan, Italy) and incubated at 37°C and 5% CO_2_ for 72 hours. Cells were washed with 1X PBS w/o Ca^2+^ and Mg^2+^ (Gibco Life Technologies, Monza, Italy) treated with 3 μM PBDE-47 or 0.0125% DMSO (control) and incubated at 37°C and 5% CO_2_ for 24 hours. The cyto- and genotoxic effects of PBDE-47 on THP-1 cell line were previously studied in our laboratory (13) and studies in this manuscript were carried out at a concentration of the toxicant in a range detected in human samples (23). The M(0) THP-1 macrophages were seeded at a concentration of 5x10^5^/mL in 100 mm cell culture dishes. Cells were washed with 1X PBS w/o Ca^2+^ and Mg^2+^ (Gibco Life Technologies, Monza, Italy), treated with 3 μM PBDE-47 or 0.0125% DMSO (control) and incubated at 37°C and 5% CO_2_ for 24 hours. Then, macrophages were polarized in M(LPS) THP-1 with 10 ng/mL LPS (*E. coli* serotype 026:B6; Sigma-Aldrich, Milan, Italy) and were incubated at 37°C and 5% CO_2_ for an additional 4 hours. Total RNAs enriched with miRNAs were extracted according to the miRNeasy Mini Kit manufacturer’s protocol (Qiagen, Milan, Italy) from five independent experiments. The *Caenorhabditis elegans* miR-39-3p spike-in exogenous control (Qiagen, Milan, Italy) was added during the extraction procedure in all samples (3.5 µl/sample at a concentration of 1.6x10^8^ copies/µl). Total RNA and miRNA concentrations were evaluated by Nanodrop quantification (NanoDrop™ One/OneC Microvolume UV-Vis Spectrophotometer, Thermo Fisher Scientific, Monza, Italy) and 250 ng of template from treated sample or control were retro-transcribed with the miScript II RT Kit (Qiagen, Milan, Italy). The cDNAs were diluted up to 200 μl and equimolar quantities of cDNA from each sample were used to prepare two different pools of cDNAs (treated or control pool, respectively). Then, 1 ng of pooled cDNA/well was analyzed by real time PCR (StepOnePlusTM Real Time PCR System, Applied Biosystems) using the miScript miRNA PCR Array Human miFinder (Qiagen, Milan, Italy, cat. MIHS-001Z) (see **Supplementary Table 1** for the list of microRNAs in the assay) and the miScript SYBR^®^ Green PCR Kit (Qiagen, Milan, Italy); each reaction was in a final volume of 25 μl/well. The real time PCR conditions were: 95°C for 15 minutes followed by 40 cycles of three step PCR: denaturation at 94°C for 15 seconds, annealing at 55°C for 30 seconds, extension at 70°C for 30 seconds. MicroRNAs expression was considered changed between the two groups (control and treated cells, respectively) if it met the following criteria: fold change <0.5 (down-regulated), fold change >2 (up-regulated), quantification cycle (CT) value<35. The CT data obtained from control and treated plates were analyzed by Gene Globe Data Analysis Center (https://geneglobe.qiagen.com/us/analyze/) using the 2^-ΔΔCT^ methods, and miRNA expression was reported as a fold change value. The normalization for calculation of the fold change was performed by the software using the automatic selection full plate normalization method. To validate the array profiling, the expression levels of miR-223 (5’TGTCAGTTTGTCAAATACCCCA Qiagen, Milan, Italy), miR-let7a (5’UGAGGUAGUAGGUUGUAUAGUU, Qiagen, Milan, Italy) and miR-155 (5’UUAAUGCUAAUCGUGAUAGGGGU, Qiagen, Milan, Italy) were analyzed in every cDNA sample used for pool preparation and normalized using cel-miR-39-3p (5’TCACCGGGTGTAAATCAGCTTG, Qiagen, Milan, Italy). The real time PCR conditions were the same reported for the array analysis. The miRNA expression was also reported in fold change using the 2^-ΔΔCT^ method.

### Pathway Enrichment Analysis

A bioinformatics analysis was carried out to study the effects of PBDE-induced miRNAs dysregulation on THP-1 macrophages molecular pathways. Specifically, a two steps analysis was performed. For the first step, starting from a single list composed of both up and down-regulated miRNAs, a set of validated miRNA-target interactions was obtained using the relationships contained in the miRTarBase (v8.0) database [mirtarbase_2018]. This database collects more than 430k experimentally validated MiRNA Target Interactions (MTIs) extracted from 11k manually curated articles. After the MTIs selection, the targets which had a greater chance of being dysregulated were considered (i.e. the sub-set of genes that interact with a significant number of miRNAs). For this aim, a graph on the extracted miRNA-target interactions was built and for the following steps, only those node targets with a higher degree than a fixed threshold were considered. **Figure 2** shows the bioinformatics workflow designed for the identification of putative miRNA-targets).

As the second step, the list of putative dysregulated genes was considered to identify the statistically relevant enriched biological pathways. For this purpose, the enrichment analysis was performed by considering the pathways collected in the Reactome platform [Reactome_2020] using the Reactome Pathway Analysis (ReactomePA) package (v1.32.0) [ReactomePA_2016], that is an R library that contains functions for automatic pathway analysis based on Reactome.

By means of the “enrich Pathway” function contained in this package, a list of pathways involved with interesting target genes was obtained. Statistical assessments of pathway enrichment were performed through the “enrichPathway” function that calculates the p-value using the hypergeometric distribution [Boyle_2004]. A correction test was also applied on p-value, using the Benjamini-Hockberg (BH) correction test [BH_1995] (beyond a cut-off of 0.05); it is a powerful adjustment method used for avoiding type I errors (false positive). Finally, the resulting list of pathways was clustered according to their biological meaning, as defined in the Reactome database, by considering the relationship between pathways within the Reactome pathway hierarchy. For each cluster, a representative pathway preserving all the target genes of the cluster was selected shows the bioinformatics workflow designed for the pathway enrichment analysis).

### THP-1 Derived Small Extracellular Vesicles (sEVs) Separation

The THP-1 monocytes were seeded at a concentration of 6x10^5^ cells/mL in 75 cm^2^ flasks and differentiated in M(0) THP-1. Then, cells were washed with micro-filtered (0.2 μm pore size) 1X PBS w/o Ca^2+^ and Mg^2+^ and incubated with 0.0125% DMSO (Control) or 3 μM PBDE-47 in RPMI medium with 10% FBS previously depleted of microvesicles by centrifugation at 118,000 g at 4°C overnight using a Beckman SW28 rotor. Thereafter, the M(0) THP-1 macrophages were differentiated in M(LPS) THP-1 for 24 hours at 37°C in 5% CO_2_. The supernatants containing DMSO derived (sEVs^DMSO^) and PBDE derived (sEVs^PBDE^) small extracellular vesicles were first centrifuged at low speed to remove cells and debris. The large EVs were then isolated by centrifugation at 10,000 x g for 30 minutes at 4°C using an Eppendorf rotor F34-6-38 and resuspended in a proper volume of 1X PBS w/o Ca^2+^ and Mg^2+^. Afterwards, sEVs were collected from the supernatants into Beckman Coulter polypropylene open top tubes *via* centrifugation at 118,000 g for 70 minutes at 4°C using a Beckman SW28 rotor. Finally, the sEVs were washed in 1X PBS w/o Ca^2+^ and Mg^2+^ and resuspended in the same buffer.

### Biochemical Characterization of THP-1 Derived sEVs

The THP-1-derived sEV protein content was measured using the BCA Protein Assay Kit (Thermo Fisher Scientific, Monza, Italy). The relative absorbance of the BCA soluble compound was measured at 562 nm using a GloMax^®^ Discover Microplate Reader (Promega, Milan, Italy).

Cell lysate and sEV samples were mixed with proper volumes of 5X loading buffer [0.25 M Tris-Cl pH 6.8, 10% SDS, 50% glycerol, 0.25 M dithiothreitol (DTT), 0.25% bromophenol blue], heated at 100°C for 5 min and loaded into 10% SDS-PAGE for electrophoretic separation. Then, proteins were blotted onto polyvinylidene fluoride (PVDF) membranes which were blocked with BSA-TBS-T solution [3% powdered with bovine serum albumin in TBST (50 mM Tris HCl pH 8.0, 150 mM NaCl with 0.05% Tween 20)] for 1 hour at room temperature, followed by primary antibody incubation overnight at 4°C. The antibodies used were: anti-Alix (clone 3A9, dil. 1:150 in 3% BSA/1X TBS-T), anti-HSP70 (clone W27 dil. 1:500 in 5% Milk/1X TBS-T), anti-Enolase and anti-βActin (clone A5 and clone AC15, respectively, dil. 1:400 in 3% BSA/1X TBS-T, Santa Cruz Biotechnology, USA). The membranes were incubated for 1 hour with the horseradish peroxidase-conjugated secondary anti-mouse or anti-rabbit antibodies according to the manufacturer’s instructions (Cell Signaling, Euroclone, Milan, Italy) and the signals were revealed using Super Signal™ Pierce™ ECL (Thermo Fisher Scientific, Monza, Italy).

### Nanoparticle Tracking Analysis (NTA) of THP-1 Derived sEVs

Nanoparticle size distribution and concentration were measured using a NanoSight NS300 (Malvern Panalytical, UK), equipped with a 488 nm laser, a high sensitivity sCMOS camera and a syringe pump, and a second 405 nm laser and a CMOS camera. The sEV samples were diluted in particle-free water (Water, HPLC grade, Sigma-Aldrich, filtered by 20 nm using Whatman Anotop filters) to generate a dilution in which 20-120 particles per frame were tracked, to obtain a concentration within the recommended measurement range (1–10x10^8^ particles/mL). Five experiment videos of 60 second duration were analyzed using NTA 3.4 Build 3.4.003 (camera level 15-16). A total of 1500 frames were examined per sample, which were captured and analyzed by applying instrument-optimized settings with a suitable detection threshold so that the observed particles were marked with red crosses and no more than 5 blue crosses were visible. Further settings, such as blur size and Max Jump Distance, were set to “automatic” and viscosity was set to that of water (0.841 - 0.844 cP). This approach applied to NTA allows interference by non-vesicle contaminants, such as nano-bubbles, insoluble salts or protein aggregates to be eliminated and, furthermore, enhances the tracking of smaller EV particles.

### Fluorescence NTA for THP-1 Derived sEVs

For F-NTA, the fluorescent THP-1 derived sEVs (F-sEVs) were produced as follows: 5x10^10^ THP-1 derived sEVs/mL were stained with 500 nM of 4-(2-[6-(dioctylamino)-2-naphthalenyl]ethenyl)-1-(3-sulfopropyl) pyridinium, DI-8-ANEPPS (Ex/Em: 467/631 nm, Thermo Fisher Scientific), previously filtered by 20 nm filters. Di-8-ANEPPS emits maximum fluorescence when bound to phospholipid membranes (such as EVs) but does not emit fluorescence when alone or bind hydrophobic protein regions. After 1 hour at room temperature, NTA analyses were carried out using NanoSight NS300 with a 500LP filter (laser wavelength 488 nm), optimized manual settings for camera level and a high flow rate for the syringe pump so that fluorescent sEVs crossed the field of vision of the main NTA screen in 5 to 10 seconds. As a negative control, we tested that the probe alone did not emit fluorescence signals with F-NTA.

### sEV miRNA Purification

Purified sEVs from 0.0125% DMSO or 3 μM PBDE-47 THP-1 M(LPS) treated cells were used for miRNA purification. The sEVs were diluted up to 200 μL with 1X PBS w/o Ca^2+^ and Mg^2+^; then the sEVs were lysed using 1 mL of QIAzol Lysis Reagent (Qiagen, Milan, Italy) and miRNA purification was performed according to the miRNeasy Serum/Plasma Kit manufacturer’s protocol (Qiagen, Milan, Italy). The steps of miRNA reverse transcription and real time PCR analysis were performed as described above. The data analysis was performed on 5 independent experiments.

### Culture of Naïve THP-1 Macrophages and sEVs

The naïve M(0) THP-1 macrophages seeded at a concentration of 6x10^5^ cells/mL in 48-well tissue cultures were incubated with sEVs^DMSO^ or sEVs^PBDE^ for 24 hours at 37°C and 5% CO_2_. The concentration of sEVs used in the cell-treatment assays were in a 1:1 or 1:2 ratios, comparable to the concentration observed in the cell culture experiments and detected by NTA analysis. Then, the cells were stimulated with 10 ng/mL LPS to polarize them in M(LPS) and incubated at 37°C and 5% CO_2_ for 4 hours. **Supplementary Figure 1** **,** reports the experimental design.

### Total RNA Preparation and Real Time Analysis From Naïve M(LPS) THP-1 Treated With sEVs

Total RNAs were extracted according to the RNeasy Mini Kit (Qiagen, Milan, Italy) manufacturer’s protocol. The cDNAs were retro-transcribed using the QuantiTect Reverse Transcription Kit (Qiagen, Milan, Italy) using 2.5 µg/reaction of RNA template. The cDNA was diluted up to 100 μl and real time analyses were performed using a StepOnePlusTM Real Time PCR System (Applied Biosystems, Monza, Italy) and SYBR Green technology. Each sample contained 50 ng of cDNA in 2X Fast SYBR Green Master Mix (Applied Biosystems, Monza, Italy) and 200 nM of specific QuantiTect primer assay (Qiagen, Milan, Italy) in a final volume of 20 μl. The primer sequences (Qiagen, Milan, Italy) used for human cytokines were: IL-6 (Interleukin 6, NM_000600) and TNF-α (Tumor necrosis factor alpha, NM_000594); the human cell motility primer sequences were: MMP7 (Matrix Metalloproteinase 7, NM_002423), MMP9 (Matrix Metalloproteinase 9, NM_004994) and MMP12 (Matrix Metalloproteinase 12, NM_002426); the housekeeping gene used in all analyses was ACT (human actin beta, NM_001101). The real time PCR conditions were: an initial denaturation at 95°C for 20 seconds, followed by 40 cycles of two-step PCR denaturation at 95°C for 15 seconds, and annealing/extension at 60°C for 30 seconds.

### Statistical Analysis

Small extracellular vesicle data analyses were performed using GraphPad Software (QuickCalcs). Statistical significance was assessed by using Mann-Whitney U-test. The Real Time Statistical analysis was performed by Wilcoxon signed rank test used for evaluating paired data. The p values < 0.05 were considered significant.

## Results

### PDBE-47 Modulates the Intracellular miRNA Profile in M(LPS) Cells

**Figure 1:**
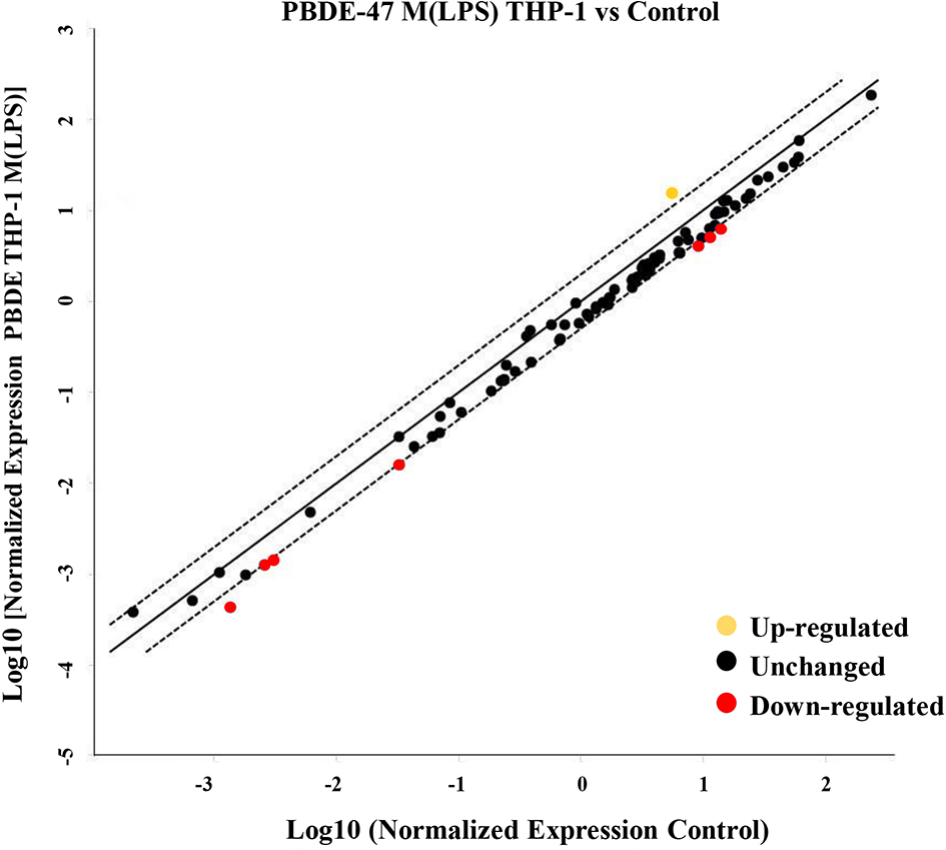
MiRNA Array. Evaluation of differential intracellular miRNA expression in cDNA derived from M(LPS) THP-1 macrophages treated with PBDE-47. The CT data obtained from control and treated plates were analyzed by Gene Globe Data Analysis Center (https://geneglobe.qiagen.com/us/analyze/) The yellow dot indicates the up-regulated miRNA (fold change >2), red and black indicate down-regulated (fold change <0.5) and unchanged miRNAs, respectively.

To evaluate the effects of PBDE-47 on the miRNA profile in M(LPS) THP-1 cells, we performed a differential miRNA expression array analysis using a commercially available microarray allowing the detection of 84 independent microRNAs as reported within **Supplementary Table 1**. According to the criteria described in the material and method section, data analysis highlighted 8 dysregulated miRNAs (see scatter plot within **Figure 1**). Specifically, PBDE-47 treatment induced an up-regulation of miR-let7a-5p (fold change= 2.1) and a down-regulation of miR-96-5p (fold change=0.4836), miR-30b-5p (fold change=0.445), miR-223-3p (fold change=0.4543), miR-99-5p (fold change=0.4616), miR-424-5p (fold change=0.4416), miR-302a-3p (fold change=0.3149) miR-130a-3p (fold change=0.4866). In **Table 1**, we report the fold change values. These modulated miRNAs (**Figure 1** and **Table 1**) were selected for further *in silico* analysis. Microarray data were validated by means of real time PCR targeting miR-223, miR-155 and miR-let-7a (**Figure 5B**).

**Table 1:** Dysregulated miRNA fold change.

Human miRNA	fold change
hsa-let-7a-5p	2.1
hsa-miR-96-Sp	0.4836
hsa-miR-30b-5p	0.445
hsa-miR-223-3p	0.4543
hsa-miR-99a-5p	0.4616
hsa-miR-424-Sp	0.4416
hsa-miR-302a-3p	0.3149
hsa-miR-130a-3p	0.4866
hsa-miR-155a-Sp	1.2

#### In-Silico Analysis of Biological Processes and Pathways by miRNA Microarray

**Figure 2:**
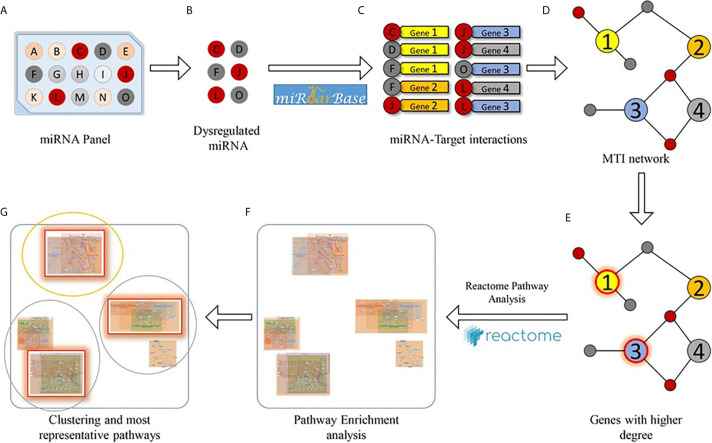
Bioinformatics workflow designed for the pathway enrichment analysis. Starting from a miRNA panel **(A)**, dysregulated miRNAs **(B)** are taken into account for obtaining a list of validated miRNA-target pairs **(C)** from the miRTarBase database. Then, the miRNA-target interactions are arranged as a network **(D)**, and genes that are targeted by at least a significant number of miRNAs **(E)** are selected for pathway enrichment analysis **(F)**. Finally, enriched pathways are clustered according to their hierarchical relationships (as defined in the Reactome database), and the most representative pathways **(G)** are given.

We used the list of the 8 PBDE-47-modulated miRNAs identified in the microarray assay (see **Table 1**) in order to discover biological pathways altered by co-expressed miRNAs, on the basis of their experimentally validated putative gene targets according to the flow chart described within **Figure 2**. Exploiting miRTarBase (http://mirtarbase.mbc.nctu.edu.tw/), a manually curated miRNA-target interactions database, we found a list of gene targets that could be affected by miRNA dysregulation. The miRNA-target interaction network highlighted 2023 putative targets which interact with a unique dysregulated miRNA. According to results reported in **Table 2**, a degree ≥ 3 miRNAs could represent a fair trade-off between the number of genes used for the pathway enrichment analysis and the reliability of putative targeted genes. Indeed, *in silico* analysis cannot ensure that validated MTIs occur in the real case of study. Following this flowchart, a set of 54 genes for the pathway enrichment analysis was selected. This set of genes was composed of 4 genes that are targeted by 5 miRNAs, 7 genes targeted by 4 miRNAs, and 43 genes targeted by 3 miRNAs.

**Table 2:** Degree of putative genes in miRNA-Target Interaction Network (MTis).

N. of targeted genes	Degree of genes(N. of miRNAs targeting the same gene)
4	5
7	4
43	3
338	2
1969	1

**Figure 3:**
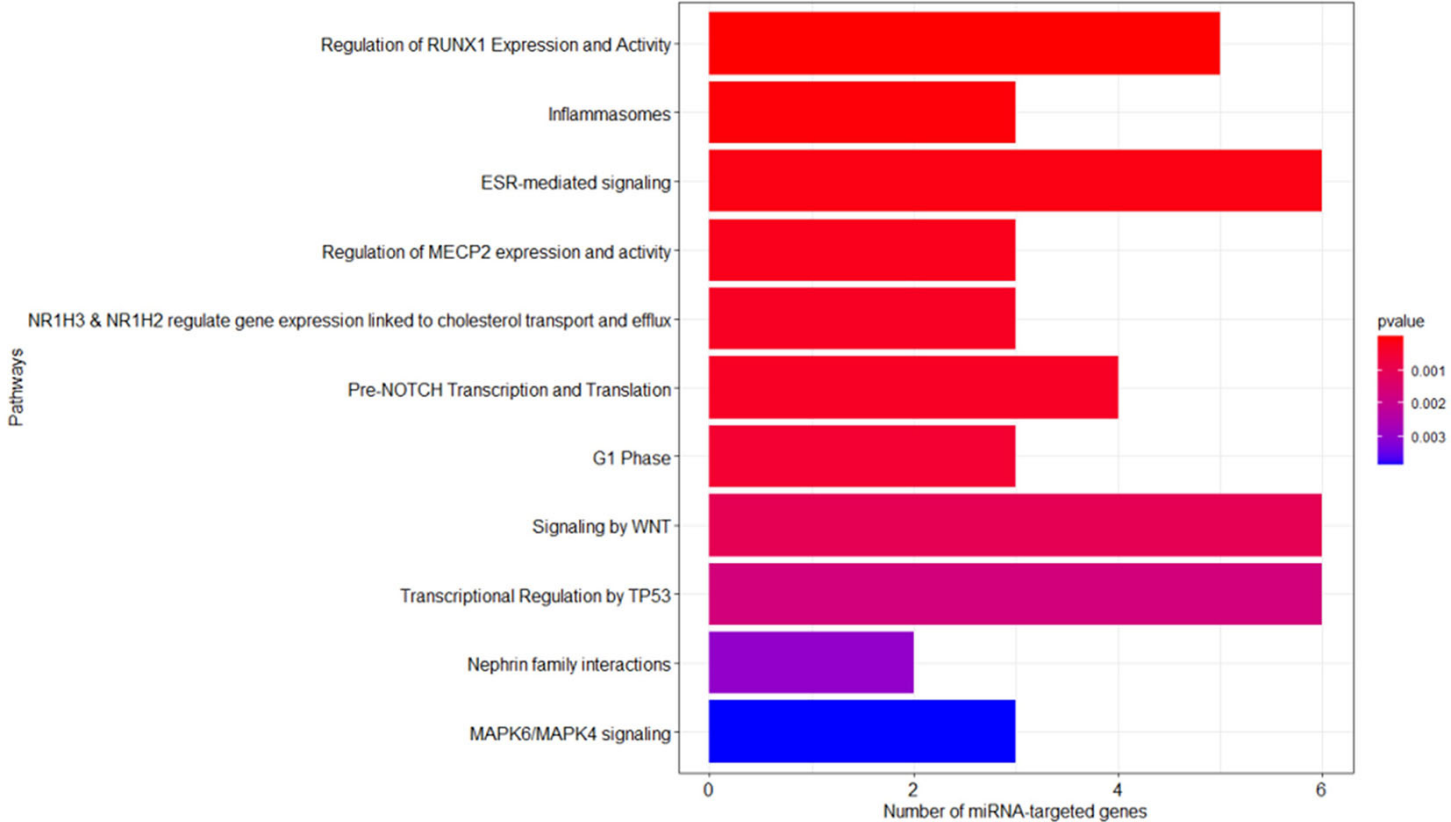
Reactome enriched pathway analysis. The bar plots show the list of pathways sorted by their statistical significance; the adopted p-value is based on hypergeometric distribution and BH correction test. The gradient of the p-value goes from red (lower p-value of 2.6348E-09) to blue (higher p-value of 3.8487E-03). The x-axis reports the number of gene targets in each pathway.

As described in the Materials and Methods section, we performed the pathway enrichment analysis by means of the “ReactomePA” library and obtained 25 statistically relevant pathways that have an adjusted p-value ≤ 0.05. Finally, we clustered these pathways according to their hierarchical relationships and shared genes, obtaining a list of 11 representative pathways of interest. We reported the complete list of representative pathways, sorted by p-value, in **Table 3**. The most statistically relevant pathway is “Regulation of RUNX1 Expression and Activity” (Reactome ID: R-HSA-8934593) with a p-value of 2.6348E-09 and 5 involved genes; whereas the second one is “Inflammasomes” (Reactome ID: R-HSA-622312) with a p-value of 4.5769E-05 and 3 involved genes. Afterwards, we found: “ESR-mediated signaling” pathway (Reactome ID: R-HSA-8939211), p-value equal to 1.2393E-04 and 6 involved genes; “Regulation of MECP2 expression and activity” pathway (Reactome ID: R-HSA-9022692), p-value equal to 2.1215E-04 and 3 involved genes; “NR1H3 & NR1H2 regulate gene expression linked to cholesterol transport and efflux” pathway (Reactome ID: R-HSA-9029569), p-value equal to 2.9917E-04 and 3 involved genes; “Pre-NOTCH Transcription and Translation” pathway (Reactome ID: R-HSA-1912408), p-value equal to 3.2003E-04 and 4 involved genes; “G1 Phase” pathway (Reactome ID: R-HSA-69236), p-value equal to 5.0123E-04 and 3 involved genes; “Signaling by WNT” pathway (Reactome ID: R-HSA-195721), p-value equal to 1.0239E-03 and 6 involved genes; “Transcriptional Regulation by TP53” pathway (Reactome ID: R-HSA-3700989), p-value equal to 1.6925E-03 and 6 involved genes; “Nephrin family interactions” pathway (Reactome ID: R-HSA-373753), p-value equal to 2.9895E-03 and 2 involved genes; “MAPK6/MAPK4 signaling” pathway (Reactome ID: R-HSA-5687128), p-value equal to 3.8487E-03 and 3 involved genes. The barplot reported within **Figure 3** shows the list of pathways sorted by their statistical significance; the gradient of the p-value goes from red (lower p-value) to blue (higher p-value). Furthermore, the x-axis reports the number of gene targets in each pathway.

**Table 3:** Representative pathways identified by the Reactome-PA database.

Reactome Pathway lD	Pathway Description	Gene Ratio (miRNA target / pathway genes)	p-value	Gene List
**R-HSA-8934593**	Regulation of RUNIX1 Expression and Activity	5/17	2.6348E-09	CCND1/CCND2/TNRC6B/AGO2/TNRC6A
**R-HSA-622312**	Inflammasomes	3/20	4.5769E-05	APP/BCL2/TXNIP
**R-HSA-8939211**	ESR-mediated signaling	6/223	1.2393E-04	CCND1/BCL2/IGF1R/TNRC6B/AGO2/TNRC6A
**R-HSA-9022692**	Regulation of MECP2 expression and activity	3/33	2.1215E-04	TNRC6B/AGO2/TNRC6A
**R-HSA-9029569**	NRIH3 & NRIH2 regulate gene expression linked to cholesterol transport and efflux	3/37	2.9917E-04	TNRC6B/AGO2/TNRC6A
**R-HSA- 1912408**	Pre-NOTCH Transcription and Translation	4/93	3.2003E-04	CCND1/TNRC6B/AGO2/TNRC6A
**R-HSA-69236**	G1 Phase	3/44	5.0123E-04	CCND1/CCND2/CDKN1A
**R-HSA- 1 95721**	Signaling by WNT	6/331	1.0239E-03	FZD6/CUL3/TNRC6B/AGO2/TNRC6A/KREME N1
**R-HSA-3700989**	Transcriptional Regulation by TP53	6/365	1.6925E-03	PRDM1/CDKNIA/TNFRSF10B/TNRC6B/AGO2/ TNRC6A
**R-HSA-373753**	Nephrin family interactions	2/23	2.9895E-03	WASL/CD2AP
**R-HSA-5687128**	MAPK6/MAP K4 signaling	3/89	3.8487E-03	TNRC6B/AGO2/TNRC6A

### Separation and Characterization of EVs Released From PDBE-47 Treated M(LPS) Cells

**Figure 4:**
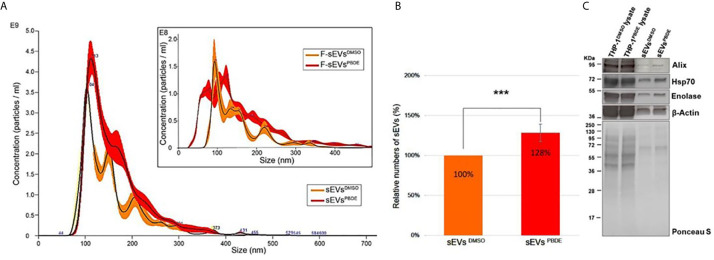
Characterization of THP-1 derived sEVs. **(A)** Nanoparticle Tracking Analysis of sEVs^DMSO^ and sEVs^PBDE^. Inset: Fluorescence NTA of sEVs^DMSO^ and sEVs^PBDE^ stained with Di-8-ANEPPS. **(B)** Relative numbers of sEVs^DMSO^ and sEVs^PBDE^. The percentage data are presented as means of four independent experiment ± SD (***p < 0.001); **(C)** Immunoblot analyses of EV biomarkers (Alix, β-Actin, Enolase, and Hsp70) in THP-1 lysates treated with DMSO or PBDE, sEVs derived from THP-1 cells treated with DMSO or PBDE. Equal amounts of total protein were loaded for cell lysates (20µg) and sEV samples (8µg). Ponceau staining is shown as a control of the total protein loaded per lane.

The conditioned culture media of M(LPS) THP-1 cells, stimulated with PBDE-47 or DMSO, were used to separate EVs according to a gold-standard method (20, 24). In this manuscript, we focused on sEV sub-populations (sEVs^DMSO^ and sEVs^PBDE^ respectively). To determine the sEV yields produced by M(LPS) THP-1 macrophages, with and without PBDE-stimulation, four experimental replicates were analyzed and compared in terms of sEV size distribution and concentration, using NTA and BCA analysis. Both types of isolated sEVs from THP-1 were in a size range of 50–200 nm in diameter (mode equal to 100 ± 10nm) and had a concentration of approximately 10^11^ particles/mL. The protein level of THP-1 derived sEVs was about 150-200 µg/mL as determined by BCA. In **Figure 4A**, sEVs^DMSO^ and sEVs^PBDE^ show an overlapping canonical size distribution, similar to the sEV profiles derived from other cell types (25). Regarding the number of tracked nanoparticles, the sEVs^PBDE^ were in a higher concentration with respect to the sEVs derived from THP-1 control cells (sEVs^DMSO^). Indeed, the statistical data analysis, carried out on four experimental replicates, indicates that PBDE stimulation significantly induced an increase in the production of sEVs of about 30% (p<0.001) in PBDE-47 treated THP-1 cells (**Figure 4B**). Therefore, we labeled both sEV samples with the Di-8-ANEPPS dye. As can be seen from the inset in **Figure 4A**, the fluorescence analysis, performed with NanoSight, confirmed the presence of plasma membrane-enclosed vesicles in both samples. Interestingly, this analysis highlighted the presence of a smaller sub-population of sEVs (with a size of about 77nm) in the F-sEVs^PBDE^, which is absent in the sEVs produced by the control cells (F-sEVs^DMSO^), which cannot be visible with NTA in light scattering readings. In addition, western blot analyses suggested that THP-1 derived sEVs were positive for specific sEV markers such as Alix, Enolase, HSP-70, and β-actin, which were all more highly enriched in both sEV samples compared to THP-1 lysates (**Figure 4C**). All of these results demonstrated that the isolated THP-1 derived sEVs possessed the characteristics of sEVs (i.e. exosomes).

#### MiR-223, miR-155, and miR-let-7a Profiling in PDBE-47 Derived sEVs

**Figure 5:**
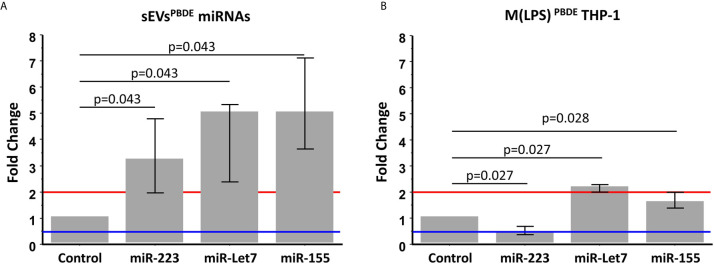
Real Time PCR analysis. Expression profile of miR-223, miR-let7a, and miR-155 in sEVs^PBDE^ **(A)** and M(LPS) ^PBDE^THP-1 cell line **(B).** Vertical bars represent medians and interquartile ranges. Red horizontal lines define a statistically significant fold increase (fold change >2), blue lines define under-regulated expression levels (fold change < 0.5). Differences from control (DMSO) were evaluated by means of Wilcoxon signed rank test.

In order to analyze the content of some key immune related miRNAs in sEVs derived from PBDE-47 treated M(LPS) cells, we decided to analyze the expression of miR-223, miR-let 7a and miR-155 in purified sEVs. By means of qPCR analysis, we compared the expression profile of the above-reported miRNAs in the sEVs^PBDE^ comparing their expression to that observed in the sEVs^DMSO^. Our analysis shows that PBDE-47 was capable of inducing a significant increase in the concentration of all three miRNAs (**Figure 5A**) (miR-223 fold change=3.2; miR-let 7a and miR-155 fold change=5).

These data highlight the remarkable difference in the intracellular level of expression of such miRNAs in the PBDE-47-treated THP-1 cells (**Figures 1**, **5B** and **Table 1**) where it has been shown that the FR induces a downregulation of miR-223 (fold change=0.4), an up-regulation of miR-let7a (fold change=2.25), and no significant differences in the levels of miR-155 (fold change=1.58). These data suggest that PBDE-47 can drive the loading of specific miRNAs in sEVs^PBDE^.

### Effect of sEVs^PBDE^ on Naïve THP-1 Macrophages

**Figure 6:**
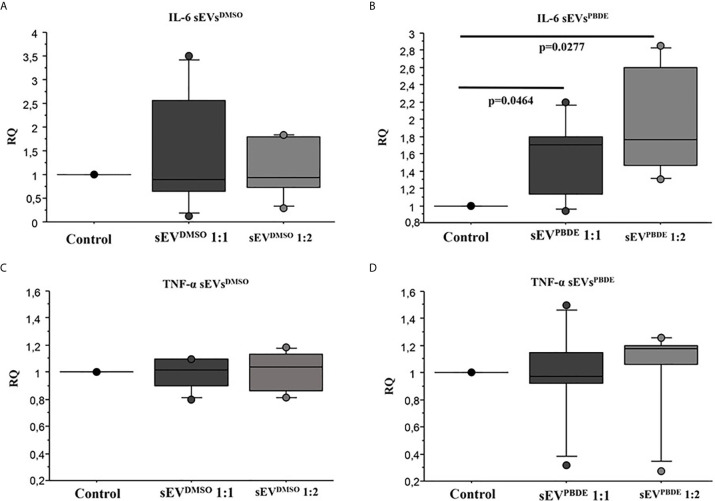
Real Time analysis of IL-6 and TNF-α cytokines in *naïve* M(LPS) THP-1 macrophages treated with sEVs. The effect of sEVs^DMSO^ on M(LPS) THP-1 macrophages **(A, C)** is comparable to control cells (treatment with LPS). Conversely, the two different concentrations of sEVs^PBDE^ **(B)** induce a statistically significant increase in IL-6 expression (p = 0.046 and p = 0.027, respectively); no significant change in TNF-α expression **(D)** was observed for both sEVs^PBDE^ concentrations used in the treatment assays. Boxplot bars indicate (from the bottom to the top) 10^th^, 25^th^, 50^th^ (median), 75^th^, and 90^th^ percentiles. Values below the 10^th^ and above the 90^th^ percentiles are plotted as circles. Statistical analysis was performed by Wilcoxon signed rank test used for evaluating not normally distributed paired data.

To study the functional effects of sEVs^PBDE^, we incubated purified sEVs^PBDE^ (at two different concentrations) with naïve M(LPS) THP-1 for 24 hours. The experimental design is summarized in **Supplementary Figure 1**. To study the effect of the sEVs^PBDE^ treatment, we decide to analyze the expression level of the pro-inflammatory cytokines IL-6 and TNF-α after LPS challenge by real time analysis. The qPCR analysis demonstrated a significant increase in IL-6 expression (**Figure 6B**, p=0.0464 and p=0.027 respectively). No differences were found in TNF-α expression (**Figure 6D**). This result demonstrated that this specific effect is due to the PBDE-47 treatment since no up-regulation of IL-6 and TNF-α mRNA was induced when sEVs^DMSO^ were used in the same experimental set-up (**Figures 6A, C**).

**Figure 7:**
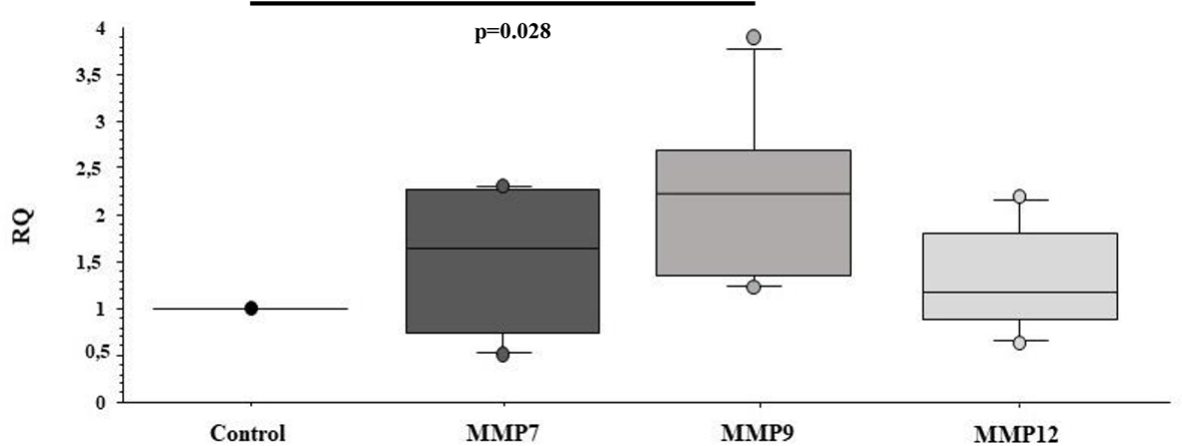
Real Time analysis of MMP genes in naïve M(LPS) THP-1 macrophages treated with sEVs. The M(LPS) THP-1 cells treated with the sEVs^PBDE^ (1:1 concentration) have a statistically significant increase in MMP9 expression (p=0.028) compared to control cells (treatment with LPS). Boxplot bars indicate (from the bottom to the top) 10^th^, 25^th^, 50^th^ (median), 75^th^, and 90^th^ percentiles. Values below the 10^th^ and above the 90^th^ percentiles are plotted as circles. Statistical analysis was performed by Wilcoxon signed rank test used for evaluating not normally distributed paired data.

Furthermore, we investigated whether sEVs^PBDE^ could have any effect on naïve M(LPS) macrophage cell motility genes, looking at the expression of a set of MMPs such as MMP7, MMP9, and MMP12. In this assay, the sEVs^PBDE^ induced a significant increase in MMP9 mRNA expression (p=0.028) (**Figure 7**) compared to control cells. No variation was observed in cells treated with sEVs^DMSO^ (data not shown).

## Discussion

Industrial development has been characterized by the introduction of a number of synthetically produced chemical compounds that have led to an ever-greater increase of novel substances released into the environment and potentially capable of affecting human health. These chemicals can enter the human body through various routes such as food consumption, breathing or adsorption. Since the immune system senses and reacts to any xenobiotic, growing attention has been paid to the role that environmental pollutants can play in the perturbation of immunological activities. In fact, in recent decades, an increase in the frequency of several immunological disorders such as immunosuppression, allergies and autoimmune diseases has been observed (26). Despite the fact that several families of brominated FRs have been listed as POPs and thus banned from production, several papers have reported the persistent presence of this class of chemicals in the environment and in animal and human tissues, suggesting a risk for human health (27).

Macrophages are important players in the innate immune system participating in both initiating the inflammatory response and maintaining immune system homeostasis (22). Macrophages are highly plastic and heterogeneous in their phenotypes and are regulated by environmental stimuli to differentiate into polarized macrophages. Based on different types of molecular signals, macrophages exhibit a continuum of phenotypes to perform different cellular and biochemical functions, from the M1 pro-inflammatory cells that contribute to infection clearance to the M2 anti-inflammatory cells that have a reparative phenotype that can contribute to the resolution phase of injury response (28). Interference with the transition from different states of macrophage differentiation may lead to pathological consequences (29).

In a previously published paper, we investigated the effects of PBDE-47 on the LPS-induced response in the PMA-differentiated macrophage cell line THP-1, demonstrating that PBDE-47 was capable of modulating macrophage and basophils activities (13).

In this manuscript, we decided to further understand the mechanism by which one of the most used FRs can modulate the innate immune response by looking at the intracellular expression profile of miRNAs as well as the biogenesis, cargo content, and activity of M(LPS) macrophage cell-derived sEVs upon PBDE-47 incubation.

MiRNAs are a major category among the noncoding RNA fraction that regulates gene expression at the post-transcriptional level. Several studies demonstrated that macrophage development and functions are regulated by the cooperative action of multiple miRNAs cooperating to control macrophage cellular pathways (30, 31). For this reason, we employed microarray analysis to study the effects of PBDE-47 treatment on the miRNA profile in M(LPS) macrophages. Using this strategy, we were able to identify a number of miRNAs modulated by the treatment, such as miR-96-5p, miR-30b-5p, miR-223-3p, miR-99-5p, miR-424-5p, miR-302a-3p, miR-130a-3p, and Let-7a-5p (**Figure 1** and **Table 1**). Our approach allowed us to perform a pathway analysis using the set of the eight above described PBDE-47-modulated miRNAs. As reported in **Figure 2**, the Reactome database was screened and pathway enrichment analysis identified 11 pathways containing genes with particular reference to the immune response and estrogen-mediated signaling (see **Figure 3** and **Table 3**).

The identification of the estrogen-mediated signaling pathway is in line with increasing evidence showing that PBDEs may act as endocrine disruptors for thyroid and reproductive hormones (32–34). Chemically, PBDEs share a structural similarity to several other environmental pollutants such as polychlorinated biphenyls (PCBs), polybrominated biphenyls (PBBs), tetrabromobisphenol A (TBBPA), and the halogenated phenyl ring of the thyroid hormone thyroxine (T4) (35). Their effects as endocrine disruptors have been documented *in vitro* (36), *in vivo* (37, 38) and in newborns and children (39, 40). Moreover, our *in silico* analysis identified the involvement of several immune-related pathways (**Figure 3**). In particular, the screening of the Reactome database highlighted that PBDE-47 treatment could modulate miRNAs involved with: 1) the regulation of the pathway for Runx 1, a factor essential for the expression of several macrophage-related genes (41); 2) the expression of genes involved in inflammasome activity; 3) the pathways of Notch signaling which is involved in the differentiation and plasticity of hematopoietic cell lineage regulating macrophage polarization (41); 4) the Wnt signaling exerting multiple roles for the monocyte-macrophage lineage that are essential for their phagocytic roles and the modulation of immune response (42, 43). **Table 3** reports the list of the specific genes targeted by the selected microRNAs. Overall, our data draw a picture in which PBDE-47 could affect the expression of several miRNAs related to the differentiation, proliferation and effector activities of the macrophages after LPS challenge, with particular reference to M1/M2 differentiation.

In addition to the cell-intrinsic modulation of miRNA-mediated regulation, several recent observations have shown increasing evidence that miRNAs can also be loaded into EVs for transport to distant cells with a phenotypic effect on recipient cells (44–46). EVs are generated as highly heterogeneous populations with respect to size and morphology and with different types of RNA cargo within them and in different amounts and proportions (44, 47, 48). Overlap in biochemical properties among different extracellular vesicle subclasses and lack of known unique markers for each subclass have made it difficult to define the RNA profiling of different subclasses. Inflammatory diseases of many etiologies can cause dramatic increase in sEV secretion (49, 50) where they do not simply reflect a random sample of cellular contents, but instead differ in both protein and miRNA content from those produced basally (51, 52). Furthermore, it has been shown that cellular activation can also alter the dynamics of EVs release by increasing the release of specific populations of vesicles (53–55). The cellular mechanism by which inflammation results in increased sEV secretion and cargo content remains to be determined.

To address the question whether incubation with PBDE-47 can have any effect on the biogenesis of sEVs and their cargo content, sEVs from PBDE-47 treated M(LPS) macrophages were purified by differential ultracentrifugation. Their number and size distribution were analyzed by NTA, showing that PBDE-47 treatment did not modify the diameter of the secreted particles but increased the number of sEVs secreted by PBDE-47 treated macrophages. Furthermore, fluorescence analysis performed using a specific probe that became strongly fluorescent upon binding to lipophilic environments such as membranes (i.e., Di-8-ANEPPS) highlighted the presence of a *de novo* population of sEVs (with a size of about 77nm) (see **Figure 4A**) within the sEVs^PBDE^ population which was absent in the sEVs secreted by the control cells, whose specific role and cargo was not further investigated in this manuscript.

To further study the effect of PBDE-47 treatment in the expression profile of sEV miRNAs, we analyzed the expression profile of a subset of the previously-studied intracellular miRNAs within the sEVs^PBDE^. Multiple *in vitro* and *in vivo* studies demonstrated that miR-223-3p, miR-155-5p, and Let 7a-5p are key regulatory players in macrophages. These miRNAs are capable of influencing several immune-related pathways targeting the secretion of IL-6 and IL-1β, two marker cytokines involved in the response against pathogens and in inflammasome activity in TLR-4 signaling induced by LPS challenge (29, 56). By means of real time PCR, we observed that the concentration of these miRNAs was strongly increased within the sEVs^PBDE^, demonstrating a PBDE-47-induced enrichment of these miRNAs in sEVs (**Figure 5A**). The trafficking of genetic materials carried by sEV cargo has been described in different cell types, fostering the core hallmarks of cell-cell intercommunication but, to the best of our knowledge, this is the first report describing a PBDE-47-mediated effect.

Several studies have identified miRNA cargoes in EVs secreted by immune cells and, conversely, EV miRNAs can be taken up by various immune cells (57). To better understand the functional role of the sEVs^PBDE^ in the cross talk between immune cells, we decided to set up a culture experiment where naïve M(LPS) macrophages were incubated with purified sEVs^PBDE^ (see **Supplementary Figure 1** for experimental set-up). After 24 hours of incubation followed by LPS stimulation, qPCR demonstrated that sEV^PBDE^ treatment is capable of exacerbating the LPS-induced pro-inflammatory response, as shown by an increase in the expression level of IL-6 mRNA (**Figures 6A–D**).

IL-6 is a key pleiotropic cytokine involved in inflammation, autoantibody production, vascular permeability, tissue regeneration, metabolism and hematopoiesis. IL-6 is produced by a large number of cells and is considered the master regulator of the production of most acute phase proteins (58). IL-6 can be considered a representative product of the pro-inflammatory M1 macrophages, but it can also elicit the development of specific cellular and humoral immune responses, including end-stage B cell differentiation, immunoglobulin secretion and T cell activation. Indeed, inflammatory cytokines such as IL-6 are potent activators of MMPs, a family of proteolytic enzymes capable of degrading all kinds of extracellular matrix proteins and involved in a number of biological processes, including tissue homeostasis, host defense and tissue repair (59). In physiological conditions, MMPs expression and activity are under tight control, whereas increased activity is usually associated with pathological conditions (60). Our data demonstrated that in the sEVs^PBDE^/M(LPS) culture assays, a significant overexpression of the MMP9 mRNA was detected (**Figure 7**). MMP9 plays important roles in immune cell function, and its proteolytic properties contribute to stimulating the immune response to initiate pathogenesis and exacerbate disease progression (61). These observations have been directly associated with the pathogenesis of chronic inflammatory diseases and cancer (62). Furthermore, a growing body of evidence demonstrates the involvement of EVs in physiological events such as the maternal‐fetal adaptations to pregnancy and immune cell interactions leading to maternal tolerance. Several papers highlighted the relevant role of EVs released by decidual macrophages in the cross talk between fetal and maternal cells during pregnancy showing that the physiological balance between M1 and M2 phenotypes is essential during pregnancy (63). In this setting, miRNAs play a key role in human placental development at different levels during gestation, where dysregulation of miRNAs in both placenta and blood has been associated with maternal exposures to several toxicants (64).

In conclusion, in this report we observed that: i) in the M(LPS) THP-1 cell line, PBDE-47 is capable of modulating the expression of a set of intracellular miRNAs mainly involved in regulating the expression of estrogen-mediated signaling and innate immune response; ii) treatment with PBDE-47 can increase the number of sEVs M(LPS) THP-1 macrophages, selecting a *de novo* population of PBDE-47-induced sEVs not found in the control sample; iii) treatment with PBDE-47 induces the overload of specific miRNAs in sEVs; iv) purified sEVs derived from PBDE-47-stimulated M(LPS) macrophages can exacerbate the LPS-induced pro-inflammatory response, inducing the overexpression of the IL-6 and the collagenase MMP9 genes.

Our study focused mainly on miRNAs and we cannot exclude that other proteins, lipids and RNA species contained in the sEVs^PBDE^ might have some effects on cellular communication differently than miRNAs thus, future studies will be required to fully understand the contribution of each component of the sEVs^PBDE^ in controlling inflammation. In conclusion, this and our previously published work suggest that an environmental pollutant such as the PBDE-47 is capable of dampening the expression of macrophage pro-inflammatory cytokines and genes involved in cell motility (51) affecting macrophage performance. Indeed, PBDE-47 is capable of modulating the macrophage intracellular miRNA profile and the biogenesis as well as cargo content of macrophage-derived sEVs, exacerbating the expression of genes involved in the LPS induced inflammatory response in naïve macrophages.

## Data Availability Statement

The original contributions presented in the study are included in the article/**Supplementary Material**. Further inquiries can be directed to the corresponding author.

## Author Contributions

VL and AL were involved in the cell culture and molecular biology assays. SP, GA, and DR were involved in the extra cellular vesicle isolation and characterization. AF, MR, and LP for the in silico analysis. AU, AB, and PC were involved for the design, analysis and reviewing of the data. FC for the statistical analysis and review of the manuscript. AL, VL, AB, and PC for writing of the manuscript. All authors contributed to the article and approved the submitted version.

## Funding

This Study was funded by the International Centre of advanced Study in Environment, ecosystem and human Health (CISAS), a multidisciplinary project on environment/health relationships funded by the Italian Ministry of Education, Universities and Research (MIUR) and approved by the Interministerial Committee for Economic Planning (CIPE) – body of the Italian Government – with Resolution no. 105/2015 of December 23, 2015.

## Conflict of Interest

The authors declare that the research was conducted in the absence of any commercial or financial relationships that could be construed as a potential conflict of interest.

## Supplementary Material

Supplementary Figure 1 Schematic representation of the experimental procedure for macrophages sEVs preparation and naïve M(LPS) THP-1 treatment with purified sEVs.
