# Supplementary figures and images for: 2,2’4,4’-Tetrabromodiphenyl Ether (PBDE-47) Modulates the Intracellular miRNA Profile, sEV Biogenesis and Their miRNA Cargo Exacerbating the LPS-Induced Pro-Inflammatory Response in THP-1 Macrophages
Source: Front Immunol. 2021 May 7;12:664534. doi: 10.3389/fimmu.2021.664534 (PMC8138315; doi:10.3389/fimmu.2021.664534)

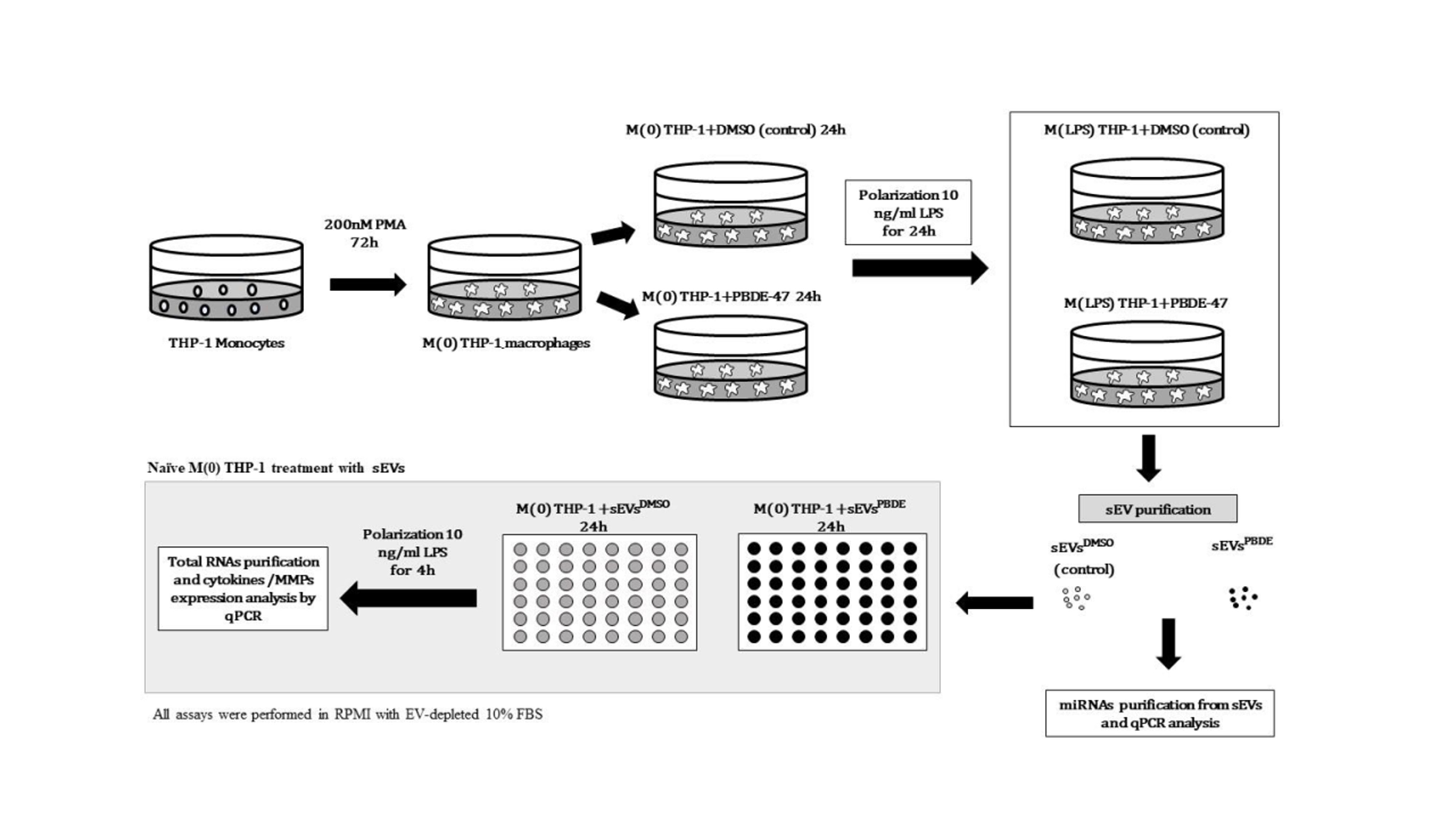

Supplement: Supplementary Figure 1 — Schematic representation of the experimental procedure for macrophages sEVs preparation and naïve M(LPS) THP-1 treatment with purified sEVs. [file Image_1.jpeg]

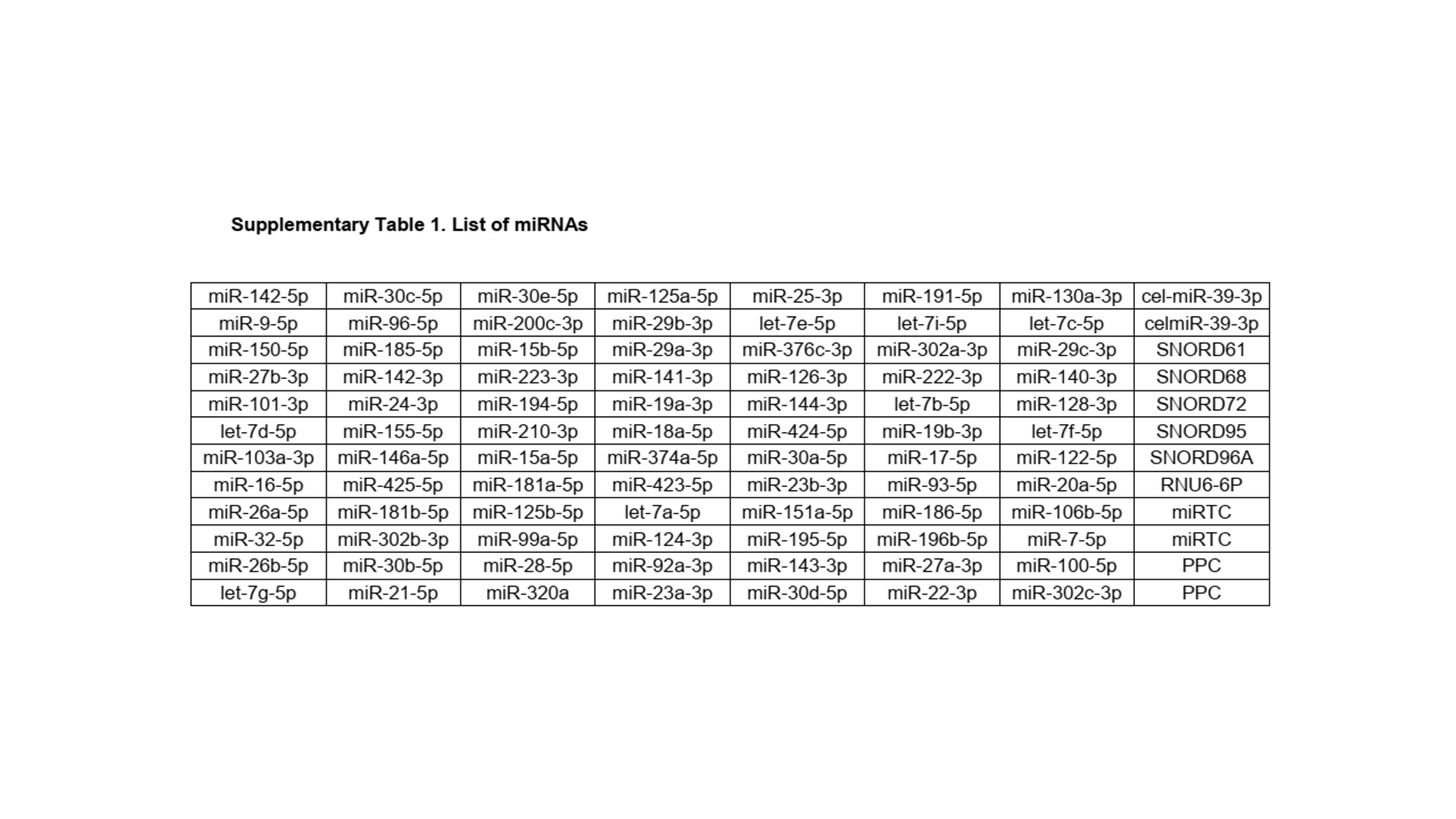

Supplement: Supplementary file 2 [file Image_2.jpeg]
